# Editorial: Neurobiological Underpinnings of Bipolar Disorder and Its Treatment

**DOI:** 10.3389/fpsyt.2021.726362

**Published:** 2021-08-06

**Authors:** Balwinder Singh, Francisco Romo-Nava, Alfredo B. Cuellar-Barboza

**Affiliations:** ^1^Department of Psychiatry and Psychology, Mayo Clinic, Rochester, MN, United States; ^2^Lindner Center of HOPE, Mason, OH, United States; ^3^Department of Psychiatry and Behavioral Neuroscience, University of Cincinnati College of Medicine, Cincinnati, OH, United States; ^4^Department of Psychiatry, Universidad Autonoma de Nuevo Leon, Monterrey, Mexico

**Keywords:** bipolar disorder, neurobiology, biomarkers, genomics, lithium

Bipolar disorder (BD) is a severe mental illness, with significant morbidity, and is a leading cause of disability. There have been significant advancements in the biological understanding of BD, with neuroimaging and genomics. This special topic on the Neurobiological Underpinnings of Bipolar Disorder and its Treatment is a mix of focused review and original findings highlighting the neurobiology of BD, and the treatment options for BD. Genomic and other biomarkers of drug response are of increasing clinical interest and have the potential to individualize treatment recommendations with greater precision for patients with BD, based on their biology. Here we provide a brief overview of the contents and highlights of the special topic.

Treatment non-compliance/non-adherence is a major problem in patients with BD, leading to higher frequency of mood relapses and impaired psychosocial functioning (1). Long-acting injectable antipsychotics are widely used to improve adherence with treatment. Li et al. in a small (*n* = 11) open-label, prospective observational study showed promising data that once monthly paliperidone palmitate (PP1M) may be beneficial in the long-term management of BD-I patients with mania predominant polarity as a monotherapy or adjunctive therapy. The PP1M treatment helped reduce the number of hospitalizations and mania/mixed episodes. The most common reported side effects with PP1M were sedation, weight gain, prolactin-related adverse events, decreased energy, and insomnia. In BD-I patients with non-adherence, this could be a promising intervention.

Neuroprogression in BD is a concept that encompasses the biological and clinical changes that, theoretically, lead to its often-devastating functional outcome. Alterations in neuroimaging and biomarkers such as neurotrophins, inflammatory and oxidative stress mediators are often regarded as evidence of neuroprogression in BD. And, despite being a thriving area of research, its molecular basis is yet to be fully demonstrated and conformed into a cohesive model (2). This Topic provides two studies that aim to investigate the links between processes previously associated with neuroprogression in BD. In a novel study, Poletti et al. explored an association between plasma inflammatory markers and Anterior Cingulate Cortex (ACC) metabolites measured using single-proton magnetic resonance spectroscopy. Glutamate (Glu), Myo-inositol (mI), and glutathione (GSH) showed higher concentrations in BD (*N* = 63) vs. Controls (*N* = 49), as opposed to NAA, a marker of neuronal health. Moreover, the authors found interesting associations between Glu and pro-inflammatory cytokine concentrations, similarly between immunoreactive IL-9 and mI, while a chemokine associated with neuron survival, CCL5, was associated with NAA levels. These findings suggest that inflammation may be a modulator of the brain metabolic changes observed in BD, for instance, by increasing oxidative stress with the consequential GSH response and eventual alteration of Glu signaling, reduced plasticity, and cell survival. This study was limited to one region of interest (ACC); it included groups with significant age differences and mostly depressed BD. Yet, it provides an important signal that merits further exploration in different mood episodes, in other brain regions, and, ideally, with a longitudinal design. In another pivotal study, Wang et al. explored the peripheric levels of neurotrophins and oxidative stress markers in BD (*N* = 97), both manic and depressed, compared to controls (*N* = 31), and controlling for smoking status and BMI. The authors found that neurotrophins, antioxidative agents, and mitochondrial DNA were decreased in BD and mitochondrial antioxidant manganese superoxide dismutase (MnSOD) was increased regardless of a mood episode. MnSOD is uniquely found in the mitochondrial matrix and is involved in oxidative stress reduction. Intriguingly, MnSOD activity was positively correlated with Brain-derived Neurotrophic factor (BDNF) levels, while mitochondrial DNA was not associated with BDNF levels. This study shows that we are a long way from scientific confirmation of neuroprogression in BD and understanding the complex interaction between processes that may be involved. Despite being readily available, peripheral markers may translate differently to brain regions, and these findings should be replicated in brain tissue.

Perhaps the ultimate goal of neuroprogression relies upon its translational value. In *Lithium and the Interplay Between Telomeres and Mitochondria in Bipolar Disorder*, Lundberg et al. explored the role of gold standard mood-stabilizing agent in cellular oxidation and aging. Alterations in the electron transport chain (ETC) in the mitochondria are believed to be the leading cause of chronic oxidative stress in BD (3). Reactive oxygen species (ROS) damage telomeres that promote cellular aging and mitochondrial dysfunction; in contrast, telomere reverse transcriptase (TERT) protects both telomere shortening and mitochondrial reaction to oxidative stress. Lithium increases ETC activity (phosphorylates ETC) in the brain and peripheral tissues, attenuates rodent mania models produced by ETC inhibition, and reverses the hyperexcitability in induced pluripotent stem cell models. Lithium also upregulates mitochondrial DNA and reduces ROS generation and oxidative stress. Similarly, lithium users show longer telomere length, and in one prospective study, lithium seems to reverse negative telomere changes observed in mania. Similar findings are described in the brain of rodent models. The authors hypothesize that lithium may directly regulate mitochondrial function and telomere length through TERT, and indirectly through the blockade of GSK3B and the activation of the Wnt canonical pathway that indirectly induces TERT transcription. Lithium's induction of mTOR would, in theory, contradict this effect and would need to be compensated for. Mitochondrial function, telomere length, and common mediators such as TERT seem to be essential avenues to develop lithium functional studies further. This review sets a further foundation for lithium's important role in BD and its neurobiological underpinnings.

The neurodevelopmental hypothesis of psychotic and affective disorders is an area of active investigation, highlighting the role of gene-environmental interactions and the impact of prenatal insults (4). Minor physical anomalies (MPAs) *may reflect basic neurobiological features underlying bipolar disorders and could be a marker of an early insult during the development*. In a systematic review, Varga et al. evaluated the association between MPAs (especially in the head and facial region) and BD. They identified four studies which met their inclusion criteria. Low grade evidence showed the number of MPAs in the BD group were significantly higher compared to healthy controls, suggesting an early insult during the neurodevelopment and provides further support to the neurodevelopmental hypothesis of BD.

We thank the authors and investigators featured in this special topic for their important and high-quality contributions.

## Author Contributions

All authors conceived and developed the presented idea and contributed to the writing of the final manuscript. All authors contributed to the article and approved the submitted version.

## Conflict of Interest

BS reports research time support from Medibio in the past (unrelated to the current study); grant support from Clinical and Translational Science (CCaTS) Small Grants Award, and Mayo Clinic. FR-N receives grant support from the National Institute of Mental Health K23 Award (K23MH120503) and from a 2017 NARSAD Young Investigator Award from the Brain and Behavior Research Foundation; is the inventor on a U.S. Patent and Trademark Office patent # 10,857,356. The remaining author declares that the research was conducted in the absence of any commercial or financial relationships that could be construed as a potential conflict of interest.

## Publisher's Note

All claims expressed in this article are solely those of the authors and do not necessarily represent those of their affiliated organizations, or those of the publisher, the editors and the reviewers. Any product that may be evaluated in this article, or claim that may be made by its manufacturer, is not guaranteed or endorsed by the publisher.
